# Student motivation to learn: is self-belief the key to transition and first year performance in an undergraduate health professions program?

**DOI:** 10.1186/s12909-019-1539-5

**Published:** 2019-04-18

**Authors:** Susan Edgar, Sandra E. Carr, Joanne Connaughton, Antonio Celenza

**Affiliations:** 1 School of Physiotherapy, The University of Notre Dame Australia, 19 Mouat St, Fremantle, Western Australia 6959 Australia; 2 Faculty of Health and Medical Sciences, The University of Western Australia, 35 Stirling Hwy, Nedlands, Western Australia 6009 Australia

**Keywords:** Motivation to learn, Admissions, Transition, Higher education, Self-belief, Curriculum

## Abstract

**Background:**

Student motivation to learn has been undervalued to date though has been identified as an area influencing student success and retention at university. The transition into university has been highlighted as a key period affecting student outcomes as well as well-being. Early identification of those students at risk may assist the transition for many students moving into higher education. Previous research has identified the Motivation and Engagement Scale – University/College (MES-UC) as a valid instrument for measuring motivation to learn in physiotherapy students. The aim of this study was to determine the relationship between a student’s motivation to learn on entry into an undergraduate physiotherapy program and their performance through first year. The relationship of admissions scores, to motivation to learn on entry, were also considered, to determine any link between these measures.

**Methods:**

An observational longitudinal study was conducted on one cohort of undergraduate physiotherapy students commencing their studies in 2015 with a response rate of 67%. Correlations were performed between admission variables and Year 1 MES-UC scoring; and between Year 1 MES-UC scoring and subsequent academic performance across first year, taking into consideration gender and age.

**Results:**

Self-belief was identified as the key dimension of motivation influencing student success in the transition into university. Results identified the link between self-belief scores on entry and academic performance in first year, including grade point average and performance in six of nine courses. Courses where there was no significant relationship were identified as curriculum areas where students may be less motivated. There was a relationship between the admissions interview and MES-UC scoring, demonstrating a link between non-cognitive selection measures and student motivation to learn on entry into the program.

**Conclusion:**

Motivation to learn and specifically self-belief with learning, may be influential in the transition into higher education. Undertaking measures of academic motivation may be useful to determine student engagement with curriculum, through identifying any link between student self-belief and performance in specific courses. Changes to curriculum based on student motivation as well as targeting early those students with reduced self-belief may improve student success, psychosocial wellbeing and retention.

## Background

There is an increasing focus on identifying factors that improve student retention at university. Completion rates and direct measures of student satisfaction and engagement have been identified as possible indicators for future performance funding in Australian higher education [1]. A review of student dropout and completion in higher education in Europe identified study success as an important issue for future policy development [2]. It was noted that research into study success impacting on completion rates and retention was limited. In a study reviewing the reasons for students leaving higher education [3], three broad factors were identified as affecting student retention: learner characteristics including motivation and cognitive abilities; external factors including the current job market and family commitments; and institutional factors including teaching quality and interactions with peers and staff. In a review of high achieving medical students’ thoughts on key factors influencing their success, four key areas were identified: motivation; learning strategies; resource management and dealing with non-academic external problems [4]. Motivation has been identified as an important contributor to student success as well as influential in determining student retention in higher education.

From a psycho-educational perspective, ‘motivation to learn’ has been described as a student’s ‘energy and drive to learn, work effectively and achieve to their potential’, in addition to the behaviours associated with this energy and drive [5]. Kusurkar et al. [6] highlighted that higher education curriculum to date has been guided predominantly by cognitive approaches rather than by motivation theory, concluding that motivation to learn has been undervalued thus far. In the Association for Medical Education in Europe (AMEE) guide ‘Motivation in medical education’ [7], it is noted that motivation is under-researched in the health sciences due to the assumption that students who enter professional courses such as medicine are highly motivated. Placing importance on ‘why’ students learn as well as ‘what’ and ‘how’, may guide educators in their teaching approaches and ultimately influence student outcomes including retention.

Multiple theories as well as dimensions or factors of motivation have been presented in the literature [6, 8–15]. Cook and Artino’s review [8] recommended additional research on academic motivation specific to health professions education and enhanced transparency with researchers identifying the ‘lens’ of motivation they are investigating, to improve clarity, application and replication. The lens or conceptual approach that has been adopted for this research is the model of academic motivation developed by Martin and represented in the Motivation and Engagement Wheel [5, 16–18]. The Motivation and Engagement Wheel [19] is a framework representative of positive and negative motivation and engagement dimensions. Positive motivation or cognitive dimensions include self-belief, valuing and learning focus. Pajares [20] noted that a person’s efficacy beliefs are linked to their effort, perseverance and resilience when completing tasks. These behavioural outcomes are also present in the adaptive behavioural dimensions of the Motivation and Engagement Wheel represented by Task Management, Planning and Persistence. Negative motivation dimensions include anxiety, failure avoidance and uncertain control. Negative engagement dimensions include self-sabotage and disengagement. The Motivation and Engagement Wheel and associated scales are supported by contemporary motivation theories [19], resulting in a broad, comprehensive instrument.

Martin designed a suite of Motivation and Engagement Scales (MES) based on the Motivation and Engagement Wheel, for respondents to contextualise to their current academic or work situation. The scales demonstrated equal validity across different domains from school to university and into the workplace [21]. The Motivation and Engagement Scale – University/College (MES-UC) has been validated for the university student population and has been found to be reliable (Cronbach’s alpha 0.78) in research conducted on undergraduate students from two Australian universities [9]. Research undertaken with the MES-UC has predominantly been conducted in the last five years with an increasing focus by researchers on utilising the instrument to both measure learner motivation and predict subsequent achievement.

Research to date has shown links between aspects of motivation as measured by the MES-UC and student typologies, adaptability and performance in their first year of university [22–24]. In a recent study focusing on the behavioural or engagement factors from the MES-UC, a relationship was seen between negative engagement in first year university students and lower semester one Grade-Point Average (GPA) for 186 undergraduate psychology students [23]. Similarly, Wurf and Croft-Piggin [24] studied the influence of MES-UC scoring early in course on first year achievement, alongside academic score on entry (via the Australian Tertiary Admission Rank or ATAR) and emotional intelligence. The MES-UC, applied at week four following commencement, was the most powerful predictor of academic achievement, greater than ATAR on entry, and accounting for 21% of the variance in the regression model. Transition into higher education, particularly post-secondary education transition, has been identified as a period of significant psychosocial adjustment with research to date acknowledging the psychological, cognitive and affective changes that student’s experience [25, 26]. Exploring how motivation to learn impacts on the first year of higher education is key to understanding the contribution of motivation to student transition, achievement and retention.

A preliminary proxy longitudinal study was conducted to review physiotherapy students’ motivation to learn, as measured by the MES-UC [27]. This study provided the first data identifying mean values for motivation dimensions for health professional students using the MES-UC. Results demonstrated the validity of the MES-UC instrument in measuring motivation and determining differences between demographic and year groups. The results, taken from 233 students, representing 82% of Physiotherapy students enrolled in a Western Australian program, identified some concerning issues including higher levels of anxiety in female students compared to males across all year groups. Disengagement from studies was also noted as a concern for male first year students, highlighting the need to investigate motivation to learn as a standalone factor influencing transition and subsequent first year performance.

The aim of this study was to determine the relationship between a student’s motivation to learn on entry into an undergraduate physiotherapy program and their progress and performance through first year, taking into consideration gender and age. The relationship of co-variables, including admissions scores and educational background, to motivation to learn on entry, were also considered.

Specifically, the following research questions were addressed:

1. What is the relationship between educational score on entry, background (school leaver versus mature age), admissions interview score and a student’s motivation to learn on entry into an undergraduate physiotherapy program, as determined by the MES-UC?
2. What is the relationship between a student’s motivation to learn on entry, as determined by the MES-UC, and subsequent first year performance? Which dimensions of motivation, as measured by the MES-UC, may enhance or negatively impact academic performance in the first year of a physiotherapy program?

Understanding the individual motivation dimensions that may influence learning and implementing appropriate interventions may improve both student motivation to learn and retention rates. Further, lower motivation levels have been associated with increased distress in medical students [28]. Facilitating improved student motivation to learn may have the added role of enhancing student wellbeing. Early identification of those students at risk may assist the transition for many students moving from secondary to higher education. Lessons learned from this study will benefit localised translation into practice, informing other institutions looking to utilise outcomes measures to identify factors influencing student success and retention.

## Methods

### Population and recruitment

This research is part of an observational longitudinal study with one cohort of undergraduate physiotherapy students from a Western Australian university, surveyed on entry into the four-year program in 2015. The cohort were subsequently surveyed every year until program completion in 2018. Participants in this study were recruited at the end of a teaching activity in week three of semester one in 2015, allowing maximal separation from assessment items to minimise any influence of assessment stress.

### Procedures

The researcher distributed hard copy participant information sheets, consent forms and surveys to students and invited them to drop their completed or non-completed surveys in a collection box at the rear of the lecture theatre following teaching activities. Students who consented to participate recorded their student number as an identifier as well as year level, age and sex. There was no incentive to participate and the researcher did not play any role in the assessment of the student cohort.

Consent to add admissions data, including educational and interview scores, to the study was sought retrospectively, as admissions scores were later deemed to be pertinent co-variables to consider. The educational score on entry is calculated from either an applicant’s ATAR, for school leavers, or their GPA of previous undergraduate studies, for mature age applicants. The interview score is calculated from performance at a semi-structured admissions interview, with questioning including aspects of an applicant’s motivation to study physiotherapy. It is an integral component within the selection process, accounting for 40% of overall admissions scoring, once applicants pass initial academic screening. Individual consent was sought for the addition of admissions data from students still enrolled in the university with a waiver of consent approved for students no longer enrolled (*n* = 7). Six students did not provide consent for the addition of admissions data. Data were sourced from existing admissions spreadsheets at the School of Physiotherapy.

### Instrument

The MES-UC is a 44-item instrument incorporating 11 dimensions of motivation, each represented by four items in the instrument, rated on a scale of 1 (strongly disagree) to 7 (strongly agree). The 11 motivation scores are grouped into four global domains. ‘Global booster thoughts’ includes scoring for self-belief, valuing and learning focus items. ‘Global booster behaviours’ includes planning, task management and persistence item scoring. For each global booster score and its individual dimensions, higher scores are more ideal. ‘Global mufflers’ represents scoring for the anxiety, failure avoidance and uncertain control items. ‘Global guzzlers’ includes item scoring from self-sabotage and disengagement dimensions. For ‘global mufflers’, ‘global guzzlers’ and their individual dimensions, lower scores are more ideal. Each of the 11 dimensions within the scale convert to a raw score out of 100.

### Data analysis

MES-UC survey results were collated in Microsoft® Excel before being transferred to IBM SPSS® Statistics Version 24.0 for analysis, with recording of all 44 items, 11 first order motivation dimensions, as well as scoring for each of the four higher order domains, per student. Admissions data including educational score on entry, interview score and background (school leaver/mature age) were also collated. The educational score was calculated by the School of Physiotherapy based on either a student’s predicted ATAR for school leavers, or a student’s previous undergraduate performance in a partially completed or completed undergraduate program for mature age students. A predicted ATAR score was calculated for each school leaver applicant by the university admissions office based on subjects studied and academic performance in the previous three semesters of school work. School leavers with a predicted ATAR of 94 or greater scored 40 out of 40, decreasing to 10 out of 40 for students scoring 85 or below. Mature age applicants who completed an undergraduate degree in a related field with a distinction/high distinction average scored 40 out of 40, decreasing to 10 out of 40 for those students having completed less than one year with a credit average. Academic results were also collated including the overall mark for every course of study undertaken and semester and year level Grade Point Average (GPA). Scatterplots were created to explore the linearity of data. The educational score was the only variable determined to not be linear in nature, due to clusters of data at extremes.

Descriptive statistics were undertaken to determine the mean, standard deviation (SD) and range of age on entry, as well as proportions of sex and background for the 2015 cohort. The mean, SD and range of admissions variables were also determined with a comparison of means conducted with a one-way ANOVA per gender and background.

Bivariate correlations were performed between the admission variables of educational score and interview score and Year 1 MES-UC scoring; and between Year 1 MES-UC scoring and subsequent performance in the program, as determined by course marks in academic units and semester and year level GPA. Spearman’s rank correlation coefficients were calculated for the educational score versus outcome variables; Pearson correlation coefficients were performed for the interview score versus outcome variables; and Point biserial correlation coefficients were calculated for the dichotomous variable background and outcome variables. A univariate analysis of variance was performed adopting a general linear model, to determine the effect of variables including each motivation factor, on subsequent performance, controlling for the variables of age and gender. Significant findings were determined by a *p*-value of less than 0.05.

## Results

### Descriptive analysis

The sample population entering first year in 2015 included 83 students, 51 (61.45%) female with an age range of 17 to 52 years (mean 19.87; SD 4.71) on entry. The cohort comprised 49 (59.04%) school leavers, with 34 (40.96%) mature age students having completed part or all a previous undergraduate degree program. Fifty-five first year physiotherapy students completed the survey representing 67% of the starting cohort with 33 (60%) females and 32 (58.18%) school leavers. The mean age was 19.91 (SD 5.32) with an age range of 17–52 years. The sample of participants were representative of the broader population of students in first year of the program.

### Admissions scores and motivation to learn

The mean educational score on entry was 32.31 (SD: 5.40) with scores ranging from 10 to 40. Females (mean: 33.48; SD: 4.17) scored higher than males (mean: 30.53; SD: 6.58) though this was not significant (*p* = 0.062). Interview scores for the cohort ranged from 20.5 to 40 (mean: 32.36; SD: 4.28). Males (mean: 33.92; SD: 4.12) scored significantly higher than females (mean: 31.30; SD: 4.11), (*p* = 0.038). Table 1 shows the relationships between admissions scores on entry and dimensions and global scores of the MES-UC as completed by students in week three of first semester. There was a significant correlation between educational score and student disengagement (*ρ* = 0.309; *p* = 0.033). The interview score correlated with scoring in three of the four global scores as well as three individual dimensions, with a further four dimensions trending towards significance. There was a negative relationship between interview score and student disengagement (*r* = − 0.406; *p* = 0.005). School leavers scored significantly higher, comparative to mature age students, in the dimensions of uncertain control (*r*_*pb*_ = 0.352; *p* = 0.008) and self-sabotage (*r*_*pb*_ = 0.275; *p* = 0.042).

**Table 1:** Correlation coefficients for admissions scores and background versus MES-UC dimension and global scores, for the 2015 cohort

		Self-belief	Learning focus	Valuing	Persistence	Planning	Task management	Anxiety	Failure avoidance	Uncertain control	Self-sabotage	Disengagement	Global booster thoughts	Global booster behaviours	Global mufflers	Global guzzlers
Interview Score	*r*	0.188	0.332	0.354	0.250	0.274	0.261	−0.010	−0.180	−0.054	− 0.272	− 0.406	0.365	0.310	− 0.100	− 0.391
	*p-value*	0.206	0.023*	0.015*	0.090	0.062	0.077	0.946	0.226	0.720	0.064	0.005*	0.012*	0.034*	0.505	0.007*
Educational score	*ρ*	0.070	−0.099	−0.172	− 0.200	−0.030	0.030	0.094	0.091	0.066	0.106	0.309	−0.073	−0.062	0.071	0.204
	*p-value*	0.637	0.505	0.242	0.174	0.842	0.841	0.524	0.537	0.655	0.472	0.033*	0.620	0.676	0.631	0.164
School leaver	*r*_*pb*_	0.018	0.031	0.189	−0.092	− 0.018	0.126	0.225	0.198	0.352	0.275	0.113	−0.105	−0.021	− 0.302	−0.248
	*p-value*	0.896	0.824	0.166	0.505	0.895	0.361	0.099	0.147	0.008*	0.042*	0.411	0.446	0.880	0.025*	0.068

*p < 0.05

### Motivation to learn and student performance in first year

Mean motivation scores and standard deviations for the cohort and per sex are presented in Table 2. Anxiety and task management were the only dimensions on the MES-UC to show a difference between genders, with females scoring significantly higher in both. When determining differences between backgrounds, two motivation dimensions had significant differences. School leavers scored higher for uncertain control (mean: 48.56; SD: 16.10) compared to mature age students (mean: 37.61; SD: 12.31; *p* = 0.008). Self-sabotage was also higher in school leavers (mean: 31.16; SD: 13.56) compared to mature age students (mean: 24.39; SD: 8.98; *p* = 0.042).

**Table 2:** Mean motivation scores (and Standard Deviations) overall and per gender

MES-UC	Mean scores (SD)	Mean scores for males (SD)	Mean scores for females (SD)	*p*-value
Self-belief	85.38 (7.78)	87.50 (8.43)	83.97 (7.10)	0.100
Valuing	88.47 (7.25)	88.64 (8.19)	88.36 (6.68)	0.893
Learning focus	90.65 (7.35)	91.05 (8.78)	90.39 (6.36)	0.751
Planning	68.02 (13.38)	67.05 (15.76)	68.67 (11.75)	0.664
Task management	78.65 (14.22)	73.68 (17.22)	81.97 (10.89)	0.033
Persistence	81.93 (9.60)	84.41 (9.18)	80.27 (9.65)	0.118
Anxiety	66.71 (18.75)	58.59 (18.28)	72.12 (17.28)	0.008
Failure avoidance	38.82 (18.73)	38.09 (21.24)	39.30 (17.19)	0.817
Uncertain control	43.98 (15.50)	42.73 (15.31)	44.82 (15.81)	0.629
Self-sabotage	28.33 (12.24)	25.45 (10.28)	30.24 (13.19)	0.157
Disengagement	26.95 (9.16)	25.09 (8.39)	28.18 (9.51)	0.222
Global booster thoughts	88.16 (5.94)	89.00 (7.08)	87.61 (5.09)	0.399
Global booster behaviours	76.25 (10.49)	75.05 (12.69)	77.06 (8.85)	0.490
Global mufflers	49.84 (14.59)	46.45 (15.02)	52.09 (14.07)	0.162
Global guzzlers	27.84 (8.90)	25.50 (7.12)	29.39 (9.70)	0.113

The mean first year GPA for the cohort was 2.29 (SD: 0.72) with no significant difference between male students (2.39; SD: 0.71) and female students (2.21; SD: 0.73; *p* = 0.368). Mature age students (mean: 2.56; SD: 0.76) had a higher first year GPA compared to school leavers (mean: 2.10; SD: 0.64) and this was significant (*p* = 0.021).

The results of the univariate analysis of variance are presented in Table 3 showing the effect of variables including each motivation factor and global score, on first year GPA, controlling for the other variables. Self-belief was the only motivation dimension to have a significant effect on first year GPA (*p* = 0.014). The effect of self-belief, controlling for gender and age, on all aspects of academic performance in first year, are presented in Table 4. There was a significant relationship between self-belief scoring on entry and academic performance in three out of four first semester courses and three out of five second semester courses.

**Table 3:** Univariate analysis of variance results showing the effect of the 11 motivation dimensions of the MES-UC and global scores, controlling for age and gender, on first year GPA

MES-UC dimensions	df	F	R squared	*p*
Self-belief	3	3.875	0.189	0.014
Valuing	3	1.898	0.102	0.142
Learning focus	3	1.901	0.102	0.141
Planning	3	1.895	0.102	0.142
Task management	3	1.960	0.105	0.132
Persistence	3	2.051	0.110	0.119
Anxiety	3	2.084	0.111	0.114
Failure avoidance	3	1.899	0.102	0.142
Uncertain control	3	2.135	0.114	0.107
Self-sabotage	3	1.999	0.107	0.126
Disengagement	3	1.916	0.103	0.139
Global booster thoughts	3	2.186	0.116	0.101
Global booster behaviours	3	1.895	0.102	0.143
Global mufflers	3	1.911	0.103	0.140
Global guzzlers	3	1.895	0.102	0.142

**Table 4:** Univariate analysis of variance results showing the effect of student self-belief, controlling for age and gender, on all first-year courses and semester GPA

First year Physiotherapy courses	df	F	R squared	*p*
Foundations of Physiotherapy Practice	3	5.28	0.241	0.003
Anatomy A	3	3.45	0.177	0.024
Behavioural Science	3	3.49	0.225	0.026
Molecular and Cell Biology	3	1.58	0.095	0.207
Semester 1 GPA	3	3.78	0.182	0.016
Anatomy B	3	3.66	0.186	0.019
Anatomy and Physiology of Body Systems	3	3.16	0.209	0.036
Movement Sciences for Physiotherapy	3	2.99	0.160	0.040
Introduction to Philosophy	3	2.63	0.294	0.080
Soft Tissue Injury Management	3	1.69	0.099	0.183
Semester 2 GPA	3	4.54	0.218	0.007

Six students exited the course by the end of first year. A comparison of means between those students who stayed and exited the program revealed no significant differences in their motivation dimensions on entry into the program.

## Discussion

The first aim of this research was to determine the relationship between students’ background and educational and interview scores on entry, and their motivation to learn as measured by the MES-UC in week three of the physiotherapy program. Of note, for this sample, there was no relationship between academic entry scores and scoring on the MES-UC, with the exception of scoring for the disengagement dimension where there was a positive relationship between academic entry scores and disengagement from learning. Given the small sample size, this may be a chance finding although disengagement from learning in first year students was also previously noted in study of a larger sample of physiotherapy students from the same university [27]. The authors postulate that the transition into the higher education learning environment as well as possible alternate aspirations for some high achieving students, including progression into the medical program, may be possible explanations for this finding.

The admissions interview correlated with three of four global scores including a positive relationship with booster thoughts and behaviours and a negative relationship with disengagement and the global behavioural score representing ‘guzzlers’. Applicants selected for interview have undertaken academic screening and have reached a threshold of academic performance deemed appropriate to complete academic tasks within the physiotherapy program. Thus, students enter with similar academic capabilities. Differentiating students that may be more motivated to learn and progress through the program, is much more difficult to determine on entry but the link between admissions interview and MES-UC does confirm that for this sample, the interview may be targeting alternate factors outside of cognitive ability. Previous research has shown a relationship between the admissions interview for this program and performance in clinical placements in Years 2–4, stronger than academic scores on entry [29]. Determining any link between academic motivation and performance though the program, including clinical performance, may be useful to determine the value of monitoring student motivation in future cohorts. Monitoring of students was highlighted as a key institutional activity to improve study success, in a report into student dropout and completion in higher education in Europe [2].

The second aim of the study was to determine any relationships between the dimensions of academic motivation and student performance, taking into consideration gender and age. Although gender differences in achievement at university have previously been identified in the literature [29–32], anxiety and task management were the only motivation dimensions to show any significant gender differences, with females scoring higher in both areas. Of note there was no link between either of these motivation dimensions and student performance so although they may have affected motivation to learn, they did not influence subsequent outcomes in first year. Anxiety towards learning may bring about enhanced task management to avoid failure [33], thus the two dimensions may have worked together to ensure satisfactory academic outcomes.

Significant relationships were found between self-belief and results in three out of four semester one courses and three out of five semester two courses. There was no relationship between the other 10 motivation factors and student performance. Self-belief, as represented on the MES-UC, denotes a ‘students’ belief and confidence in their ability to understand or to do well in their university/college studies, to meet challenges they face, and to perform to the best of their ability’ [31]. This definition of self-belief is congruous with ‘self-efficacy’, where students make cognitive judgements of their capabilities [34]. Zajacova et al. [35] further termed self-efficacy in the academic context as ‘academic self-efficacy’, referring to a student’s confidence in their ability to complete a particular learning activity or task. Motivation to learn and self-efficacy have an integrated or co-dependent relationship as determined by contemporary motivation theories. In the expectancy value theory of motivation, individuals are more likely to engage in tasks where they have higher self-efficacy or belief about their actions and the likely outcomes that will follow [13]. In Bandura’s social cognitive theory [11, 36, 37], the perceived importance of the task is central to motivation with self-efficacy underpinning a person’s beliefs about their personal competence. Pajares [20] noted that a person’s efficacy beliefs are linked to their effort, perseverance and resilience when completing tasks and further highlighted the link between self-efficacy and emotional reactions with decreased self-efficacy leading to stress, depression and/or reduced problem-solving abilities. Likewise, Zajacova et al. [35] found academic self-efficacy and stress to be negatively correlated. Thus, tapping into a student’s self-efficacy or self-belief may be the key to both improving performance and decreasing stress. Targeting students with lowered self-belief on entry into higher education and providing appropriate intervention, may result in improved student outcomes including retention.

It is important for universities to understand how student self-efficacy interacts with institutional characteristics as this may ultimately influence retention rates. Self-belief scoring was linked to performance in certain first year courses comparative to other courses. Two out of three of the courses where there was no link between self-belief scoring and student performance, were not delivered by the physiotherapy program, with the third course since undergoing substantial changes due to student feedback on curriculum provided through traditional course review processes. This may indicate that measuring academic motivation may be useful to assist curriculum review and feedback. Curriculum development based on motivation theory needs further investigation though Turner [38] identified the role of developing experiences through the higher education journey based around control, success and improvement, to foster self-belief in students.

The initial transition into university has been highlighted as a key period to provide intervention, with a review of Australian higher education students from eight institutions, undertaken mid-year, revealing that just over a third of first year students reported having difficulty getting motivated to study [39]. Similarly, a review of psychosocial adjustment of first year college students in the U.S. noted a significant decline in psychological, cognitive and affective well-being in first semester [25]. The decline plateaued in second semester with the researchers noting that identification and intervention in first semester was paramount. It appears that the key time to measure and implement any intervention to enhance motivation to learn is within the first six months of first year.

The authors acknowledge the limitations of this study, including the small sample size of study participants from one cohort of physiotherapy students from a Western Australian university. Further, this study involved the use of a self-reporting instrument applied at one time point, to determine motivation to learn, based on a framework developed by A.J Martin, supported by contemporary models of motivation theory [19]. A preliminary proxy longitudinal study determined the validity of this instrument for the population tested [27].

The literature points towards context and institution-specific research as being the key to understanding the complex construct of student motivation [8, 40]. The value of lessons learned from a local study to produce benefits through localised translation into practice, cannot be underestimated. Thus, this study reported on findings from investigating motivation to learn, specific to a physiotherapy program, considering the social context and interplay between a student’s motivation including their academic self-efficacy and the role of localised curriculum, specific to the learner. It is important to note that although the sample size for this study was not large, moderate effect sizes were shown in the correlation findings. Further research will review students’ change in motivation over time, as measured by the MES-UC, as well as relationships between academic motivation and performance throughout Years 2–4 of the program, including clinical performance. This may assist with planning the timing of any proposed intervention to enhance academic motivation during the degree program.

## Conclusion

In a sample from one physiotherapy undergraduate program, there is a relationship between the admissions interview score on entry and motivation to learn, as measured by the MES-UC, applied at week three of the program. Self-belief, though not related to other admissions elements, was linked to academic performance in the transition into university, as measured by first year results. Motivation to learn and specifically self-belief with learning, may be influential in the transition into higher education. Consideration of individualised follow-up for students with lowered motivation levels on entry, may be appropriate. Motivation measures, such as the MES-UC, may be pertinent to determine student engagement with curriculum, ensuring that experiences in first year programs foster student self-efficacy with learning.

## Abbreviations

ATAR: Australian Tertiary Admission Rank
GPA: Grade-Point Average
MES-UC: Motivation and Engagement Scale – University/College
